# Morphological Variation and Its Environmental Correlates in the Taihangshan Swelled-Vented Frog across the Qinling Mountains

**DOI:** 10.3390/ani12182328

**Published:** 2022-09-07

**Authors:** Lei Fu, Xiaoyi Wang, Shengnan Yang, Chunlin Li, Junhua Hu

**Affiliations:** 1 School of Resources and Environmental Engineering, Anhui University, Hefei 230601, China; 2 Chengdu Institute of Biology, Chinese Academy of Sciences, Chengdu 610041, China; 3 Anhui Province Key Laboratory of Wetland Ecosystem Protection and Restoration, Anhui University, Hefei 230601, China

**Keywords:** adaptive potential, Bergmann’s rule, environmental change, *Feirana taihangnica*, morphological traits, skeletochronology

## Abstract

**Simple Summary:**

Amphibians have weak dispersal abilities and are sensitive to environmental changes, resulting in their disproportionately high risk of extinction, with many species’ populations rapidly declining. Therefore, it is critical for amphibian conservation to understand their adaptive potential by exploring how amphibians respond to environmental changes based on morphological variations. Our results showed that morphological traits of *Feirana taihangnica* significantly differed among ages. Along with the increase in annual mean temperature, snout-vent length showed an anti-hump trend, indicating no support for Bergmann’s rule. Mean ultraviolet-B of the highest and lowest months were positively and negatively correlated with head width, thigh length and tibia width, respectively. The present study can help understand the effects of environmental changes on morphological variations of this mountain frog species and its adaptive potential, providing important implications for species conservation.

**Abstract:**

The Taihangshan swelled-vented frog (*Feirana taihangnica*), an endemic species to the Qinling Mountains, central China, has experienced a dramatic population decline over the last few decades. The aim of this work was to quantify morphological variation in *F. taihangnica* across the Qinling Mountains and examine environmental correlates of this variation of morphological traits. We implemented a hierarchical partitioning to estimate the independent contribution of each environmental variable on morphological variations. Temperature seasonality was the greatest contributor in variations of snout-vent length (SVL) and head width, and ultraviolet-B (UV-B) radiation of the lowest month was the most influential on both thigh length and tibia width. Then, we used generalized additive models to analyze the relationship between each environmental factor and morphological trait variations. Along the increasing of annual mean temperature, SVL decreased firstly and then increased, indicating no support for Bergmann’s rule. Furthermore, SVL was negatively correlated with annual precipitation, while positively with temperature seasonality. The mean UV-B of the highest and lowest months was positively and negatively correlated with head width, thigh length and tibia width, respectively. The results of this study help us to understand adaptive potential of this mountain frog species via morphological variations in the light of environmental changes.

## 1. Introduction

Environmental changes can affect animals’ physiology [1], phenology [2,3], morphology [4], distribution [5] and life-history strategies [6,7]. The environmental variations across a species’ distribution range provide an opportunity to assess the adaptive potential of animals to environmental changes [4,8,9]. Notably, amphibians are particularly vulnerable to environmental stresses and changes due to their highly permeable skin, unshelled eggs, and low dispersal abilities [10]. Previous studies have explained how amphibians respond to environmental changes through different strategies, such as niche shift [11,12,13], phenological changes [2,14], and variations in morphological traits [7,15,16]. Morphological variations that, in particular, reflect a strong link between an organism’s fitness and processes in the community and ecosystem, can help us understand how amphibians respond adaptively to environmental stresses and changes [4].

Along environmental gradients, morphological traits of amphibians may exhibit large variations [4,17]. The tendency in which increasing body size of organisms is associated with colder climates is a well-known ecogeographical rule, i.e., Bergmann’s rule [18,19]. Although there is no consensus on the generalization of Bergmann’s clines in amphibians [18,20], some studies found that decreasing temperature and precipitation promoted larger body size [7,21]. Furthermore, ultraviolet-B (UV-B) radiation can have diverse effects on amphibians [22]. For example, high UV-B radiation would damage DNA and lead to decreasing survival rate of larvae [22,23], whereas some anurans may decrease head width, vertebrae length and femur length due to inadequate levels of UV-B radiation [24].

The Taihangshan swelled-vented frog (*Feirana taihangnica*) is endemic to the Qinling Mountains, central China, occupying an elevational range from 500 to 1700 m [25]. Hu and Jiang suggest that interspecific competition may prevent the southward expansion of *F. taihangnica* at the broad spatial scale [26]. As a stream-dwelling frog, it is particularly sensitive to habitat alterations due to its communal breeding behavior, strong fidelity to oviposition and long larval period [27]. Human disturbances (e.g., habitat destruction and exploitation) have led to population decline in recent years, highlighting the need for the conservation of this species [27,28]. This species is now classified as ‘vulnerable’ in the Red List of China’s Vertebrates [28].

Here, we explore how environmental changes (i.e., temperature, precipitation and UV-B radiation variables) affect morphological variations in *F. taihangnica*. Specifically, we aim to (1) examine the effects of temperature and precipitation conditions on body size of *F. taihangnica*, (2) characterize how morphological traits respond to the influences of UV-B radiation conditions, and (3) test the applicability of Bergmann’s rule in *F. taihangnica*. This study would help to understand the adaptive potential of mountain frog species via morphological variations in light of environmental changes.

## 2. Materials and Methods

### 2.1. Data of Morphological Traits and Age Estimation

**Figure 1:**
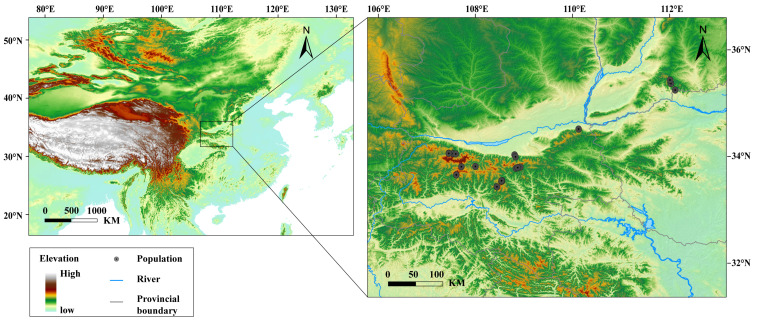
Geographic distribution of the studied populations for *Feriana taihangnica*.

We investigated 16 sampling sites for *F. taihangnica* across the Qinling Mountains (Figure 1, Table S1). For each sampled individual, we measured four morphological traits, including snout-vent length (SVL), head width, thigh length and tibia width, with electronic digital calipers (YB5001B, Kraftwelle Company, Hangzhou, China) to the nearest 0.02 mm (Table S2). Representing body size, SVL is a critical functional trait influencing a host of other species traits (e.g., reproductive performance, competitive ability, and extinction risk) [6,29]. Head width is usually associated with prey shape and food acquisition [30,31]. We also measured the thigh length and tibia width, which have been proved to be related to locomotor performance such as burrowing, jumping and swimming [32]. All specimens were deposited in the Herpetological Museum, Chengdu Institute of Biology (CIB), Chinese Academy of Sciences (CAS) (Table S1).

The ages of each sampled individual were estimated by skeletochronology [15,33]. We first removed the surrounding skin and muscle tissue of all digits and washed digits in running water for two hours and decalcified them in 5% nitric acid [15]. Then, we washed phalanges in running water overnight and stained them with Ehrlich’s haematoxylin for 75 min. The stained bones were dehydrated through ethanol concentrations of 70%, 80%, 90% and 100% for one hour in each stage and infiltrated through successive concentrations of paraffin for one hour in the thermostat (50 °C). Next, bones were embedded in small paraffin blocks. Then, we used a microtome (KD-202, KEDEE Company, Jinhua, China) to obtain a 13 μm thick cross-section of the phalanx and mounted it on glass slides. We identified the number of lines of arrested growth (LAG) from mid-diaphyseal sections under a LEITZ dialux 40 microscope and photographed with a digital camera (Motic BA300, Motic Company, Chengdu, China) mounted on a Moticam 2006 light microscope at 400× magnification. Each LAG was formed when frogs experienced a hibernation period, which means that the number of LAGs reflected actual age. We also counted the incomplete rings in the outer margin of bones because individuals were collected prior to the hibernation period. The number of LAGs was counted independently by two experienced people to control observer error. A total of 69 individuals were estimated for age, with measurements of the four morphological traits.

### 2.2. Environmental Predictors

We used bioclimatic and UV-B variables as environmental predictors to explore variation in morphological traits of *F. taihangnica* [34,35]. To avoid high collinearity among bioclimatic variables, we examined their correlations and excluded certain variables using Person’s correlation tests (|r| > 0.75) (Figure S1, Table S3) [36]. We retained seven bioclimatic variables, including annual mean temperature, mean monthly temperature range, temperature seasonality, min temperature of the coldest month, annual precipitation, precipitation of the driest month and precipitation seasonality. Bioclimatic data were obtained from WorldClim v2 [35]. We also obtained the mean UV-B of the highest and lowest months, respectively, from the glUV dataset v1 [34]. These variables were found to be influential for anurans’ survival and can help to understand the impacts of environmental change on morphological variations in anurans [22,37]. We extracted environmental predictors for the sampling sites using ArcGIS 10.2 (ESRI, Redlands, CA, USA).

### 2.3. Data Analyses

To reduce the dimensionality of environmental predictors, we implemented a principal component analysis (PCA) based on a correlation matrix. We selected the first two principal components (PCs), i.e., PC1 and PC2, with the threshold of eigenvalue > 1.0, which explained 84.14% of the total variance (Table S4). Then, we used linear mixed models (LMMs) to assess the effects of environmental change along PCs (PC1 and PC2) on morphological traits. PC1, PC2 were regarded as fixed variables and sampling site as a random factor, age and sex were considered as covariates to control its impact on morphological traits. We also used the R package ‘hier.part’ to implement a hierarchical partitioning to estimate the independent contribution of each environmental variable on morphological variations [7,38]. Next, we used generalized additive models (GAMs) to examine environmental effects on variation in morphological traits [39]. Environmental predictors were assigned as fixed smooth term and age was added as a random effect smooth. All statistical analyses were carried out in R 4.1.2 (R Core Team 2021, Vienna, Austria).

## 3. Results

**Figure 2:**
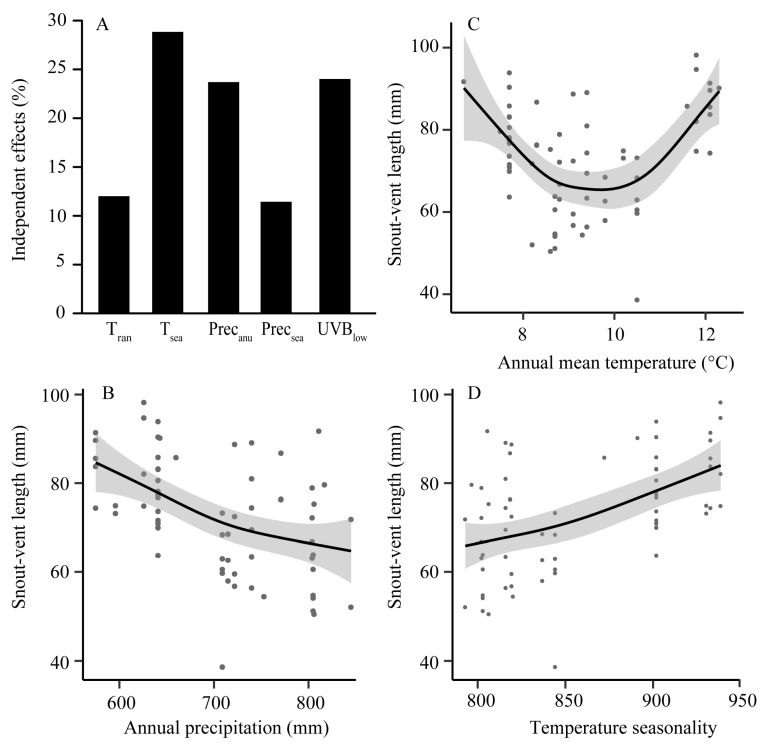
Estimated environmental effects on snout-vent length (SVL). (**A**) Independent contribution for each environmental effect (in percentage) on SVL of *Feirana taihangnica* based on hierarchical partitioning. T_ran_: mean monthly temperature range, T_sea_: temperature seasonality, Prec_anu_: annual precipitation, Prec_sea_: precipitation seasonality, and UVB_low_: mean UV-B of the lowest month; (**B**–**D**) variation in SVL along different environmental gradients. Illustrations are predicted smoothed regression lines with confidence intervals from generalized additive mixed-effects models.

The ages of the sampled individuals ranged from one to eight years, with 68% (47 specimens) being between four and six. The age class of five years old had the largest number of individuals, and age distribution showed the population increased first and then decreased (Figure S2). Principal component axes 1 (PC1) and 2 (PC2) accounted for 63.01% and 21.13% of the total variance in the environmental predictors, respectively. PC1 was correlated with mean monthly temperature range, temperature seasonality, annual precipitation, precipitation seasonality and mean UV-B of the lowest month; PC2 mainly responded to annual mean temperature (Table S4). All morphological traits significantly differed among ages and environmental predictors along PC1 (Table 1). Hierarchical partitioning revealed that temperature seasonality was the greatest contributor to both SVL (Figure 2A) and head width (Table S5), and mean UV-B of the lowest month was the most influential on both thigh length and tibia width (Table S5).

The four morphological traits were mostly correlated significantly with environmental predictors (Table S6). SVL was negatively correlated with annual precipitation (R^2^ = 0.34, *p* < 0.001; Figure 2B), while positively with temperature seasonality (R^2^ = 0.36, *p* = 0.004; Figure 2D). Along the gradient of annual mean temperature, SVL displayed an inverse hump-shaped pattern (decreased gradually, and then increased after the bottom at intermediate temperatures; R^2^ = 0.39, *p* < 0.001; Figure 2C).

**Figure 3:**
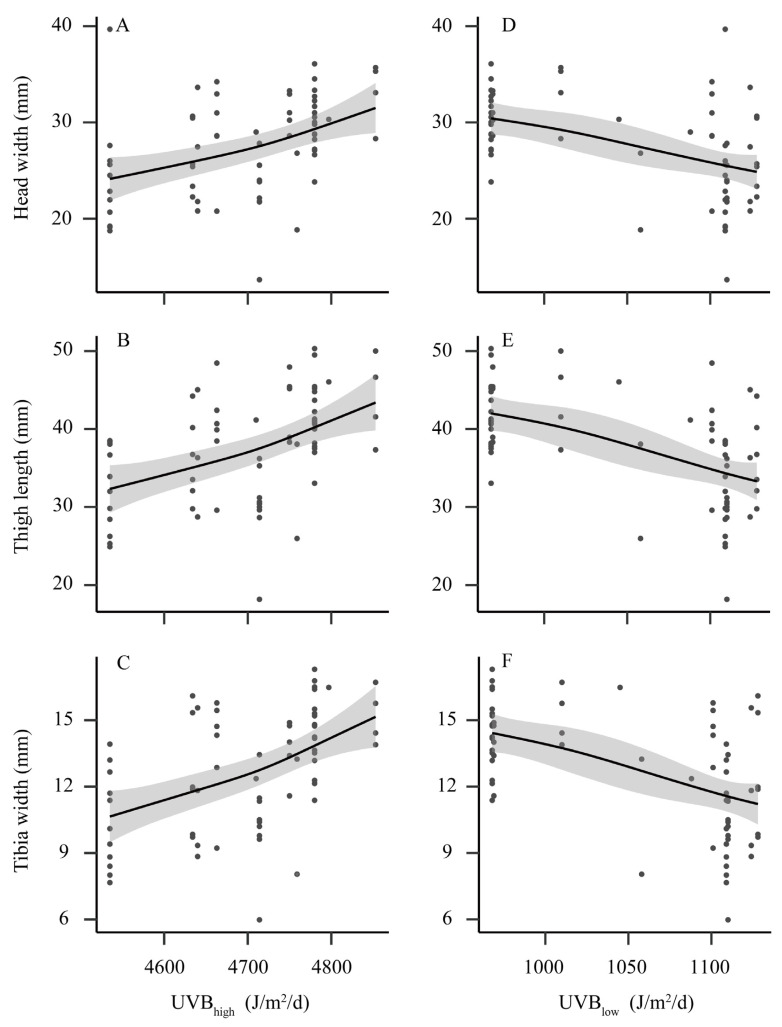
Variation in morphological traits along the gradient of ultraviolet-B (UV-B) radiation. (**A**–**C**) the gradient of mean UV-B of the highest month (UVB_high_); (**D**–**F**) the gradient of mean UV-B of the lowest month (UVB_low_). Illustrations are predicted smoothed regression lines with confidence intervals from generalized additive mixed-effects models.

Significant positive trends were detected between head width (R^2^ = 0.33, *p* < 0.001), thigh length (R^2^ = 0.36, *p* < 0.001), tibia width (R^2^ = 0.45, *p* < 0.001) and mean UV-B of the highest month (Figure 3A–C). On the other hand, mean UV-B of the lowest month was negatively correlated with head width (R^2^ = 0.38, *p* = 0.002), thigh length (R^2^ = 0.45, *p* < 0.001) and tibia width (R^2^ = 0.45, *p* < 0.001) (Figure 3D–F).

## 4. Discussion

This study focused on the question of how different morphological traits of *F. taihangnica* respond to changes in environmental conditions. This species is highly vulnerable to environmental change because of poor dispersal ability, strict habitat and slow growth [25,27]. We found that different environmental predictors have discriminative effects on morphological variations of *F. taihangnica*, hinting adaptive potential of this mountain frog species under changing environmental conditions.

Age is an important life-history trait and often reflects the growth trajectory of given individuals [33]. The lifespan of *F. taihangnica* was eight years, while the number of young and old individuals was less than the middle-aged group (Figure S2), suggesting stabilization of population growth. The variation of body size is considered a species’ adaptation to local environments [40]. It is well known that endothermic organisms may have smaller body sizes in warmer regions than their relatives in cold environments because of thermoregulation [19,41]. The generalization of Bergmann’s rule to amphibians has been widely controversial, with some species exhibiting body size clines consistent with the rule, whereas other species do not show the expected pattern [18,29,41]. Our results showed that SVL of *F. taihangnica* decreased firstly and then increased along the increase of annual temperature (Figure 2C), indicating that Bergmann’s rule may be not present in this mountain frog species. This would be reasonable, as larger body sizes in warm environments can result in greater heat dissipation and make other size-related benefits (e.g., competition and predation) [17,41].

The observed negative relationship between annual precipitation and SVL of *F. taihangnica* was in accordance with the prediction from the water availability hypothesis, suggesting that larger body sizes in amphibians would appear in relatively drier environments since a lower surface/mass ratio can reduce the loss of water [42,43]. Moreover, among the environmental factors, temperature seasonality acted as the greatest contributor to SVL (Figure 2A), and there was a positive association between them (Figure 2D; Table S6). One possible explanation is that larger individuals can be more adapted to the seasonally fluctuating temperature environments where animals experience a long duration of food deprivation [17]. Larger individuals may have higher resistance to starvation because energy reserves increase faster than energy depletion as body size increases [17,44]. Hence, for *F. taihangnica*, a larger body size is a positive tactic for adapting to colder, drier and more seasonally fluctuating temperature environments. Furthermore, a larger body size usually has a larger hind limb length with higher locomotor performance that improves survival in harsher environments [45]. The strategy of growing bigger also increases reproductive opportunities for males [46].

For many amphibian species, even though both temperatures and precipitation are crucial for their distribution ranges and morphological characteristics [4,26], some other environmental variables, such as UV-B radiation, can be powerful explanatory variables [22]. Our results indicated that high values of UV-B radiation in the highest month had positive effects on the growth of *F. taihangnica*, whereas the mean UV-B of the lowest month showed contrary trends (Figure 3). The cost of repairing cellular damage, producing protective pigments or behaviorally avoiding harmful UV-B radiation regions may retard individual growth in the presence of low levels of UV-B [47]. Conversely, relatively high UV-B exposure can promote calcitriol secretion and show preferential allometric skeletal development of components [24]. Notably, for *F. taihangnica*, the resultant increased head width, thigh length and tibia width can improve predation and locomotor abilities [31,32]. UV-B radiation may influence embryo survival and development in amphibians [23,24]. Stream-dwelling anurans have the characteristics of delayed maturation, slow growth and long lifespan when compared to arboreal and terrestrial species [27,48]. Understanding how environmental changes modified the morphological traits of amphibians is important for their conservation [4]. We found positive effects of temperature seasonality and negative effects of annual precipitation on body size. In addition, our study demonstrated that low UV-B exposure can cause negative effects on the growth of *F. taihangnica*, whereas high UV-B exposure shows positive effects. Overall, this study showed different effects of temperature, precipitation, and UV-B radiation variables on morphological variations in *F. taihangnica.* These findings would be helpful for the conservation of this mountain frog species. To better understand the impacts of environmental changes on morphological variations of this species, future studies clearly need to consider some other local factors (e.g., food availability, interspecific interaction) [16,49], and explore species responses to future environmental changes (e.g., climate change, land-use change) [36,50,51].

## 5. Conclusions

In total, we sampled 69 individuals of the amphibian *Feirana taihangnica* across the species distribution range in China to examine the effects of temperature, precipitation and Ultraviolet-B radiation on morphological traits of the species. We found positive effects of temperature seasonality and negative effects of annual precipitation on body size. In addition, low UV-B exposure can cause negative effects on the growth of *F. taihangnica*, whereas high UV-B exposure shows positive effects. Further work on the effects of other factors on morphological trait variations should focus on more specific variables and verify whether the variability of morphological traits is caused by phenotypic plasticity alone or in combination with genetic adaptation [4].

## Figures and Tables

**Table 1:** Results of linear mixed models (LMMs), assessing the effects of environmental predictors along PCs (PC1 and PC2) on morphological traits (i.e., snout-vent length, head width, thigh length, and tibia width), age and sex were considered as covariates.

Traits	Factors	Random Effect		Fixed Effect	
		LRT	*p*	SE	F
Snout-vent length	Population	1.87	0.17		
	Sex			2.37	6.51 *
	Age			0.88	14.93 ***
	PC1			0.64	6.63 *
	PC2			1.44	<0.01
Head width	Population	0.16	0.69		
	Sex			0.98	4.72 *
	Age			0.35	12.71 ***
	PC1			0.21	13.20 *
	PC2			0.47	0.29
Thigh length	Population	0.90	0.34		
	Sex			1.31	2.69
	Age			0.48	10.77 **
	PC1			0.32	11.58 *
	PC2			0.73	0.89
Tibia width	Population	1.96	0.16		
	Sex			0.50	0.76
	Age			0.19	14.47 ***
	PC1			0.13	8.35 *
	PC2			0.30	0.26

LRT: log likelihood ratio test; SE: standard error; Levels of significance are shown as: *, *p* < 0.05; **, *p* < 0.01; ***, *p* < 0.001.

## Data Availability

The data presented in this study are available on request from the corresponding author. The data are not publicly available due to privacy or ethical restrictions.

## Supplementary Materials

The following supporting information can be downloaded at: https://www.mdpi.com/article/10.3390/ani12182328/s1, Table S1: Summary of location information and sample size of study populations of *Feirana taihangnica*; Table S2: Summary of four morphological characteristics measured for each specimen of *Feirana taihangnica*; Table S3: Environmental variables compiled to depict environment gradients for *Feirana taihangnica*; Table S4: The first two principal components (eigenvalue > 1.0) and factor loadings of principal component analysis; Table S5: Independent contribution for each environmental effect (in percentage) on morphological traits; Table S6: Results of generalized additive models (GAM), assessing environmental effects on morphological traits (i.e., snout-vent length, head width, thigh length, tibia width); Figure S1: Pearson’s correlation tests for bioclimatic variables; Figure S2: The lifespan of *Feirana taihangnica* and the number of individuals in each age class.
