# Supplementary material for: Morphological Variation and Its Environmental Correlates in the Taihangshan Swelled-Vented Frog across the Qinling Mountains
Source: Animals (Basel). 2022 Sep 7;12(18):2328. doi: 10.3390/ani12182328 (PMC9495075; doi:10.3390/ani12182328)
Supplement: Supplementary file 1 [file animals-12-02328-s001.zip › animals-1880560-supplementary.pdf]

Morphological variation and its environmental correlates in the Taihangshan swelled-vented frog across the Qinling Mountains

Lei Fu[1, 2], Xiaoyi Wang[2], Shengnan Yang[2], Chunlin Li[1, 3*], Junhua Hu[2*]

[1]School of Resources and Environmental Engineering, Anhui University, Hefei 230601, China

[2]Chengdu Institute of Biology, Chinese Academy of Sciences, Chengdu 610041, China

[3]Anhui Province Key Laboratory of Wetland Ecosystem Protection and Restoration, Anhui University, Hefei 230601, China.

*Corresponding authors:

Chunlin Li, E-mail: lichunlin1985@163.com; Tel. 18226601609; Address: No.111, Jiulong Road, Hefei 230601, China

Junhua Hu, E-mail: hujh@cib.ac.cn; Tel. +86 28 82890256; Address: No. 9 Section 4, Renmin Nan Road, Chengdu 610041, China

Table S1. Summary of location information and sample size of study populations of *Feirana taihangnica*.

Locality	Latitude (° N)	Longitude (° E)	Region	Sample size	Voucher number
Ningshan1	33.55	108.55	Shaanxi	12	2006072216, 2006072242, 2006072243, 2006072244, 2006072245, 2010080802, 2010080803, 2010080804, 2010080805, 2010080806, 2010080808, 2010080810
Ningshan2	33.43	108.45	Shaanxi	2	2010080812, 2010080815
Zhashui1	33.80	108.84	Shaanxi	3	2010080502, 2010080503, 2010080506
Zhashui2	33.78	108.84	Shaanxi	1	2010080501
Zhashui3	33.80	108.93	Shaanxi	1	2010080705
Zhouzhi1	33.82	108.00	Shaanxi	6	2010081101, 2010081102, 2010081104, 2010081105, 2010081106, 2010081108
Zhouzhi2	33.66	107.61	Shaanxi	1	2010081203
Chang'an1	34.00	108.83	Shaanxi	1	2010080402
Chang'an2	34.03	108.81	Shaanxi	1	2010080401
Taibai1	34.06	107.46	Shaanxi	4	2008082213, 2008082214, 2008082215, 2008082216
Taibai2	34.05	107.60	Shaanxi	6	2008082201, 2008082202, 2008082203, 2008082205, 2008082206, 2008082207
Taibai3	34.06	107.54	Shaanxi	3	2008082208, 2008082209, 2008082210
Tongguan	34.52	110.13	Shaanxi	4	2011051314, 2011051315, 2011051317, 2011051318
Jiyuan	35.25	112.12	Henan	5	2010041901, 2010041902, 2010041903, 2010041905, 2010041906
Yuanqu	35.37	112.03	Shanxi	2	2006071520, 2006071538
Qinshui	35.44	112.01	Shanxi	17	2006071290, 2006071291, 2006071296, 2006071297, 2006071299, 2006071301, 2006071501, 2006071502, 2006071506, 2006071518, 2006071527, 2006071528, 2006071531, 2006071532, 2006071535, 2006071536, 2006071537

Table S2. Summary of four morphological characteristics measured for each specimen of *Feirana taihangnica*.

Morphological characteristic	Summary	Important function
Snout-vent length	From tip of the snout to the end of vent	important trait correlating with many physiological and biochemical processes and determining life history and ecological niche [49].
Head width	Maximum width of the head (right behind the eye)	prey shape and food acquisition [30].
Thigh length	Distance between the midpart of the urostyle and the knee	locomotor performance: burrowing, jumping, climbing, swimming and so on [32].
Tibia width	Maximum width of the tibia (midpart)	

Table S3. Environmental variables compiled to depict environment gradients for *Feirana taihangnica*.

Abbreviation	Environmental variables	Included in the study
UVB3	Mean UV-B of the highest month [34]	**Yes, UVB_{high}**
UVB4	Mean UV-B of the lowest month [34]	**Yes, UVB_{low}**
bio01	Annual mean temperature [35]	**Yes, T_{anu}**
bio02	Mean monthly temperature range [35]	**Yes, T_{ran}**
bio03	Isothermality [35]	No
bio04	Temperature seasonality [35]	**Yes, T_{sea}**
bio05	Max temperature of the warmest month [35]	No
bio06	Min temperature of the coldest month [35]	**Yes, T_{col}**
bio07	Temperature annual range [35]	No
bio08	Mean temperature of the wettest quarter [35]	No
bio09	Mean temperature of the driest quarter [35]	No
bio10	Mean temperature of the warmest quarter [35]	No
bio11	Mean temperature of the coldest quarter [35]	No
bio12	Annual precipitation [35]	**Yes, $Prec_{anu}$**
bio13	Precipitation of the wettest month [35]	No
bio14	Precipitation of the driest month [35]	**Yes, $Prec_{dri}$**
bio15	Precipitation seasonality [35]	**Yes, $Prec_{sea}$**
bio16	Precipitation of the wettest quarter [35]	No
bio17	Precipitation of the driest quarter [35]	No
bio18	Precipitation of the warmest quarter [35]	No
bio19	Precipitation of the coldest quarter [35]	No

Table S4. The first two principal components (eigenvalue > 1.0) and factor loadings of principal component analysis.

Variables	PC1	PC2
Annual mean temperature	0.070	0.982
Mean monthly temperature range	0.931	0.028
Temperature seasonality	0.936	0.233
Min temperature of the coldest month	-0.631	0.770
Annual precipitation	0.930	-0.318
Precipitation of the driest month	-0.525	-0.283
Precipitation seasonality	0.914	-0.240
Mean UV-B of the highest month	0.825	0.138
Mean UV-B of the lowest month	-0.933	0.177
Eigenvalue	5.67	1.90
Total variance explained (%)	63.01	21.13

Table S5. Independent contribution for each environmental effect (in percentage) on morphological traits. T_{ran}, mean monthly temperature range; T_{sea}, temperature seasonality; $Prec_{anu}$, annual precipitation; $Prec_{sea}$, precipitation seasonality, and UVB_{low}, mean UV-B of the lowest month.

Morphological traits	T_{ran}	T_{sea}	$Prec_{anu}$	$Prec_{sea}$	UVB_{low}
Snout-vent length	12.01	**28.84**	23.70	11.43	24.02
Head width	12.89	**28.91**	20.53	10.74	26.93
Thigh length	12.27	25.02	18.73	12.25	**31.73**
Tibia width	12.94	26.38	20.60	11.38	**28.70**

Table S6. Results of generalized additive models (GAM), assessing environmental effects on morphological traits (i.e., snout-vent length, head width, thigh length, tibia width).

Morphological traits	Environmental variables	df	F	*P*
Snout-vent length	Annual mean temperature	3.354	6.353	<0.001
	Mean monthly temperature range	3.845	3.382	0.021
	Temperature seasonality	5.617	3.638	0.004
	Min temperature of the coldest month	5.792	3.143	0.010
	Annual precipitation	1.000	16.280	<0.001
	Precipitation of the driest month	1.000	1.834	0.181
	Precipitation seasonality	1.000	5.262	0.025
	Mean UV-B of the highest month	3.428	6.345	<0.001
	Mean UV-B of the lowest month	6.875	4.454	<0.001
Head width	Annual mean temperature	3.123	5.386	0.002
	Mean monthly temperature range	3.551	3.690	0.016
	Temperature seasonality	1.000	18.340	<0.001
	Min temperature of the coldest month	2.710	4.067	0.001
	Annual precipitation	1.000	16.750	<0.001
	Precipitation of the driest month	1.000	2.239	0.140
	Precipitation seasonality	1.000	7.415	0.008
	Mean UV-B of the highest month	1.000	16.410	<0.001
	Mean UV-B of the lowest month	6.258	4.026	0.002
Thigh length	Annual mean temperature	5.447	5.596	<0.001
	Mean monthly temperature range	3.699	4.738	0.004
	Temperature seasonality	5.308	4.937	<0.001
	Min temperature of the coldest month	3.075	5.765	0.002

	Annual precipitation	1.000	20.460	<0.001
	Precipitation of the driest month	1.000	2.781	0.101
	Precipitation seasonality	1.000	10.590	0.002
	Mean UV-B of the highest month	1.002	21.090	<0.001
	Mean UV-B of the lowest month	7.269	5.066	<0.001
	Annual mean temperature	6.281	3.314	0.007
	Mean monthly temperature range	1.000	11.410	0.001
	Temperature seasonality	5.624	5.072	<0.001
	Min temperature of the coldest month	2.615	5.651	0.002
Tibia width	Annual precipitation	5.568	5.369	<0.001
	Precipitation of the driest month	1.000	3.527	0.065
	Precipitation seasonality	1.000	10.990	0.002
	Mean UV-B of the highest month	4.628	6.761	<0.001
	Mean UV-B of the lowest month	7.220	4.623	<0.001

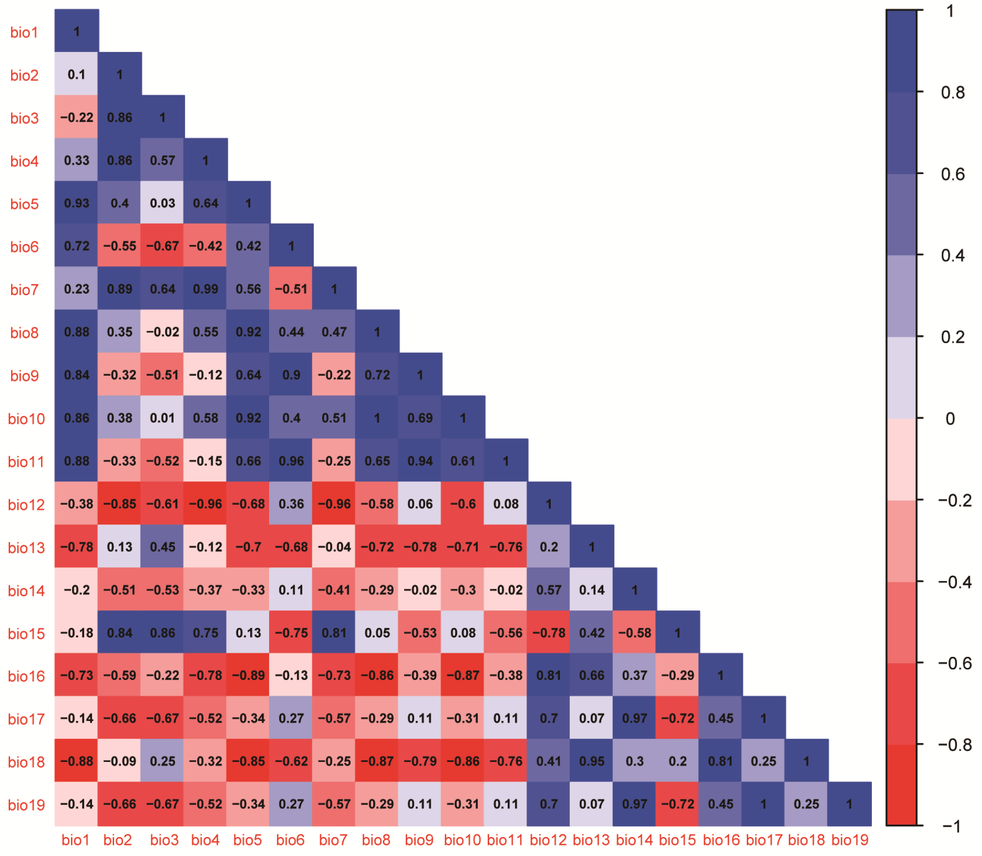

Figure S1. Pearson's correlation tests for bioclimatic variables. See Table S3 for the abbreviations of environmental variables.

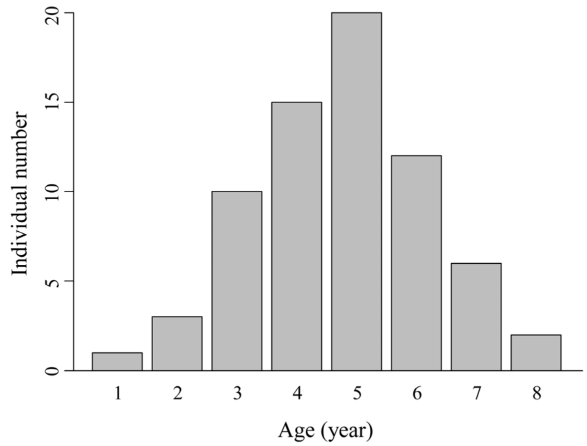

Figure S2. The lifespan of *Feirana taihangnica* and the number of individuals in each age class.
